# CT-Derived Anatomical Factors Associated with Operative Time and Hearing in Endoscopic Stapes Surgery: A Retrospective Cohort Study

**DOI:** 10.3390/diagnostics16162512

**Published:** 2026-08-09

**Authors:** Shao-An Lu, Yu-Hsuan Kuo, Hsing-Mei Wu

**Affiliations:** 1 Otorhinolaryngology Head and Neck Surgery Department, Shin Kong Wu Ho-Su Memorial Hospital, Taipei 111045, Taiwan; linch768@gmail.com (S.-A.L.); ssheisverytall@gmail.com (Y.-H.K.); 2 Medical School, Fu-Jen Catholic University, New Taipei City 242062, Taiwan

**Keywords:** otosclerosis, endoscopic stapes surgery, external auditory canal, audiological outcome

## Abstract

**Background/Objectives:** The objective of this study is to evaluate anatomical factors associated with operative time and audiological outcomes in endoscopic stapes surgery, with particular focus on quantitative temporal bone CT measurements. **Methods:** A retrospective review was conducted of 40 patients (51 ears) who underwent exclusive endoscopic stapes surgery between 1 April 2014 and 31 March 2023 by a single surgeon. Preoperative and postoperative audiometric data, operative time, and high-resolution temporal bone CT measurements—including scutum angle and external auditory canal (EAC) cross-sectional area—were analyzed. Correlation analysis was performed to assess associations with operative time and air–bone gap (ABG) closure. Receiver operating characteristic (ROC) analysis was conducted as an exploratory assessment to identify anatomical thresholds associated with prolonged operative time. Additional analyses were performed to evaluate the potential influence of surgical period on operative time. **Results**: Significant improvements were observed in postoperative air conduction, bone conduction, and ABG (all *p* < 0.0001). Mean ABG closure was 21.4 dB, and 74.5% of ears achieved postoperative ABG ≤ 10 dB. Operative time demonstrated a strong positive correlation with scutum angle (r = 0.8968, *p* < 0.0001) and a moderate negative correlation with EAC area (r = −0.5759, *p* < 0.0001). No significant correlation was identified between CT measurements and audiological outcomes. Exploratory ROC analysis suggested that an EAC area < 0.1922 cm^2^ and scutum angle > 71.4° were associated with longer-than-median operative times. No significant difference in operative time was observed among the three predefined surgical periods, and linear regression analysis demonstrated no significant association between surgical period and operative time. **Conclusions**: Endoscopic stapes surgery provides favorable audiological outcomes. While preoperative CT-based anatomical parameters were not associated with hearing results, narrower EAC dimensions and larger scutum angles were associated with increased operative time under the endoscopic approach. These findings may facilitate preoperative surgical planning by identifying patients who are likely to present greater technical difficulty during endoscopic stapes surgery. However, the proposed CT thresholds should be regarded as exploratory findings and require prospective external validation before routine clinical implementation.

## 1. Introduction

The first modern stapes surgery was developed in 1956 for the management of patients with otosclerosis [1]. Since then, surgical techniques have evolved because of the advent of operating microscopes. Stapes surgery can primarily be classified as stapedotomy or stapedectomy. Stapedotomy involves the creation of a small hole in the stapes footplate, whereas stapedectomy entails partial or complete removal of the stapes footplate [2]. The focus of both procedures is to address ossicular chain abnormalities and improve conductive or mixed hearing loss resulting from otosclerosis.

Endoscopic approaches to ear surgery were first developed in 1967, researchers were focused on improving visualization [3]. Over time, the transcanal endoscopic approach developed, and it has evolved to include tympanoplasty, cholesteatoma excision, ossiculoplasty, and stapes surgery [4,5,6]. In such procedures, using an operating microscope enables surgeons to manipulate instruments with both hands and provides stability. However, a microscope has narrow operating field. This limitation, which occurs because of visual axis restrictions, necessitates the removal of tissue for adequate visibility [7].

The endoscopic approach has been proposed as an alternative method for stapes surgery. It offers wider operative field, enhanced magnification, reduced trauma to bone and soft tissues, and fewer complications [8,9]. Moreover, the endoscopic approach often involves shorter operation times than does the microscopic approach [10]. Recent advances in endoscopic ear surgery have further highlighted its ability to provide panoramic visualization of the middle ear, particularly around the oval window, facial recess, and retrotympanum, thereby reducing the need for excessive bony removal in appropriately selected patients. These anatomical advantages have recently been comprehensively reviewed by Alicandri-Ciufelli et al., supporting the expanding role of endoscopic techniques in otologic surgery [11].

Studies have reported comparable audiological outcomes between the microscopic and endoscopic stapes surgeries. However, the endoscopic approach is particularly advantageous in cases of otosclerosis because it provides clearer visualization of the anatomy around the stapes [12].

Although previous studies have described the anatomical advantages of endoscopic stapes surgery and compared its audiological outcomes with those of microscopic surgery, quantitative evaluation of preoperative CT-derived anatomical parameters in relation to operative complexity under an exclusively endoscopic approach remains limited. Therefore, the present study investigated whether temporal bone CT findings could predict operative time and audiological outcomes in endoscopic stapes surgery.

## 2. Materials and Methods

This study was conducted from 1 April 2014 to 31 March 2023. Patients presenting with suspected stapes disease who underwent endoscopic stapes surgery were included in the study. All operations were performed by the same surgeon. Ethical approval for this study was obtained (Shin Kong Wu Ho-Su Memorial Hospital IRB number: T-20250206, approval date: 26 June 2026), and all patients in this study signed the informed consent in accordance with institutional ethical standards. And all data were fully anonymized.

Data on patient demographic characteristics, preoperative pure tone audiometry (PTA), postoperative PTA (at least 2 months postsurgery), preoperative air–bone gap (ABG), postoperative ABG, ABG closure, operative time (the interval from the initial skin incision to completion of wound dressing), and high-resolution temporal bone computed tomography (CT) findings were collected. The average PTA value was calculated using the frequencies of 500, 1000, 2000, and 4000 Hz. High-resolution temporal bone CT is routinely performed before stapes surgery to evaluate middle-ear anatomy at our institution, identify congenital anomalies or alternative causes of conductive hearing loss, assess the facial nerve course, and facilitate preoperative surgical planning. Patients who did not have confirmed stapes disease, lacked temporal bone CT images, or did not undergo stapes surgery were excluded from the study.

**Figure 1:**
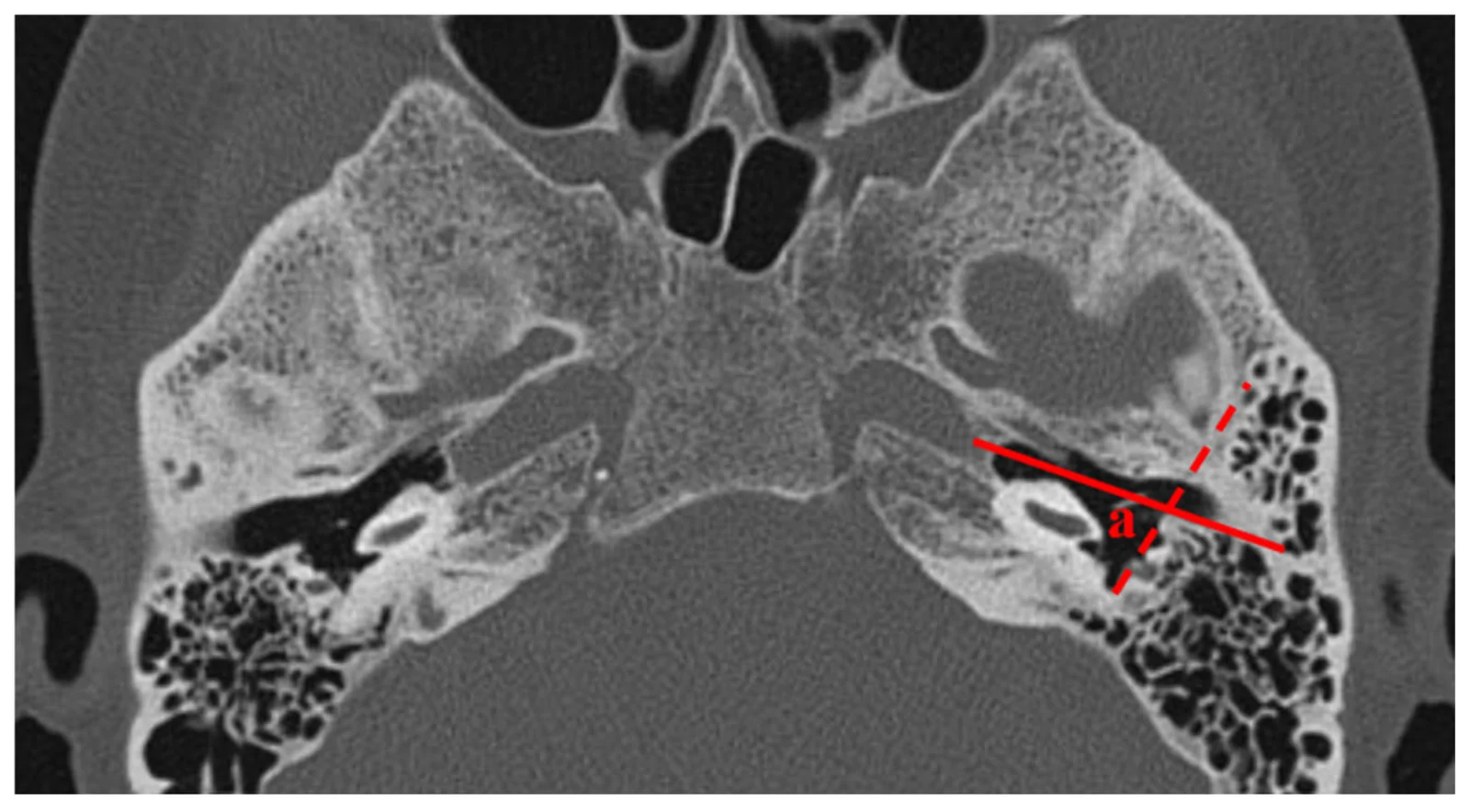
Axial view of high-resolution temporal bone computed tomography (CT) image revealing the scutum angle (a). The solid line represents the extension of the external auditory canal (EAC) wall, and the dotted line indicates the tympanic cavity wall. The angle formed between these two lines is the scutum angle.

**Figure 2:**
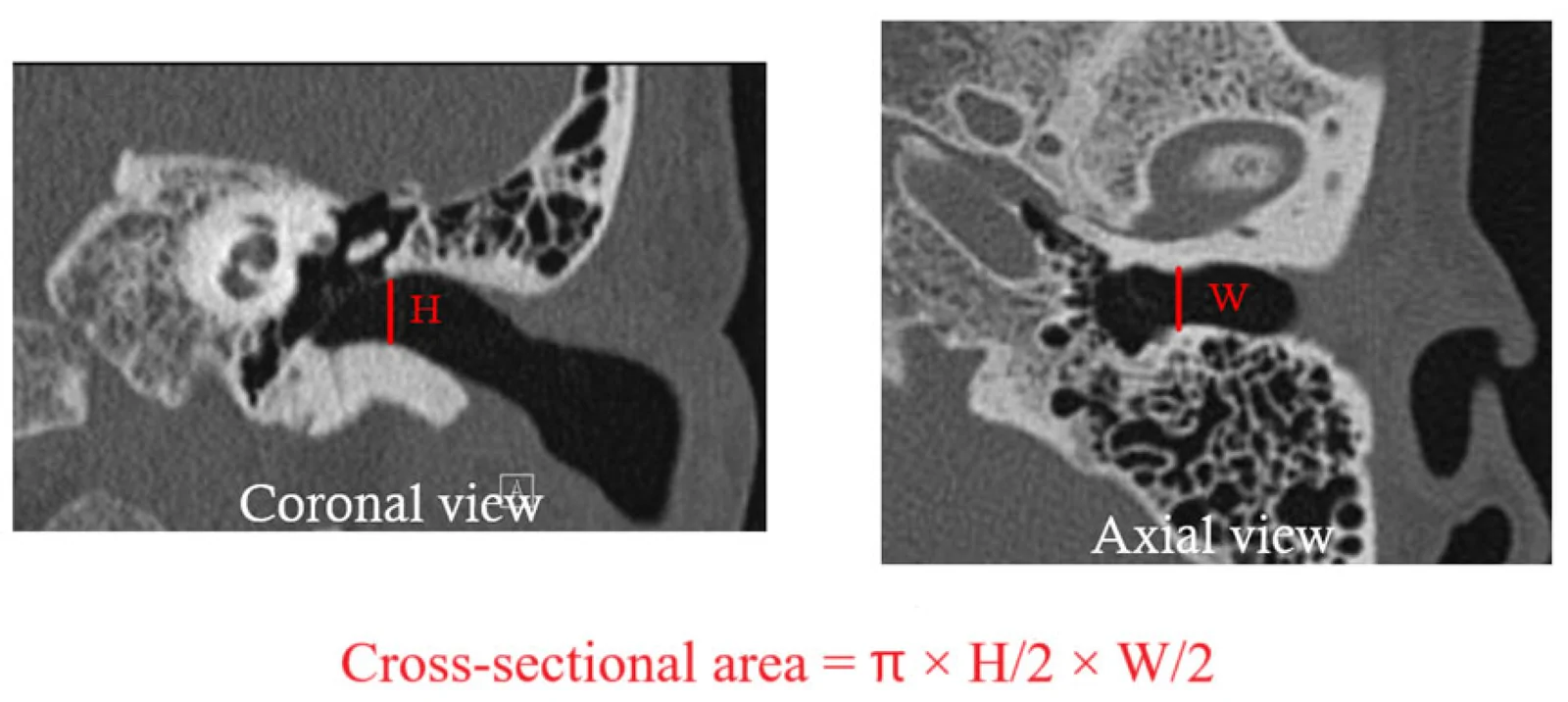
Measurement of the external auditory canal (EAC) isthmus dimensions on high-resolution temporal bone CT. The height (H) and width (W) of the EAC isthmus were used to calculate its cross-sectional area as follows: π × H/2 × W/2.

All temporal bone CT measurements were performed retrospectively by a single observer using a standardized measurement protocol. Temporal bone CT scans provided measurements of the scutum angle and the width and height of external auditory canal (EAC). The scutum is a sharp, bony spur formed by the superior wall of EAC and the lateral wall of tympanic cavity. The scutum angle is angle between the extension line of EAC wall and tympanic cavity wall in the axial view (Figure 1). The height and width of EAC isthmus were used to estimate the cross-sectional area, which was calculated as π × height/2 × width/2 (Figure 2).

This study investigated factors that predict surgical outcomes, such as operative time and ABG closure, which was calculated as the difference between preoperative and postoperative ABGs. Statistical analysis was performed by MedCalc^®^ Statistical Software version 22.009 (MedCalc Software Ltd., Ostend, Belgium; 2023), with the significance level set at *p* < 0.05. The paired *t*-test was used to compare preoperative and postoperative PTA results. Categorical variables were analyzed using Spearman’s correlation coefficient. Correlation coefficients were classified as indicating moderate (0.4 to 0.59 or −0.59 to −0.4), strong (0.6 to 0.79 or −0.79 to −0.6), and very strong (0.8 to 1 or −1 to −0.8) correlation. To identify potential predictive variables, receiver operating characteristic (ROC) curve analysis was used to calculate cutoff values for candidate features indicating the suitability of endoscopic stapes surgery. Each feature was categorized as having acceptable (0.7 to 0.8), excellent (0.8 to 0.9), or outstanding (0.9 to 1) discrimination. To evaluate the potential influence of increasing surgical experience, additional analyses were performed by dividing the study period into three predefined intervals (2014–2017, 2017–2020, and 2020–2023). Operative time was compared among these periods using one-way analysis of variance, and linear regression analysis was performed to assess temporal trends.

## 3. Results

**Figure 3:**
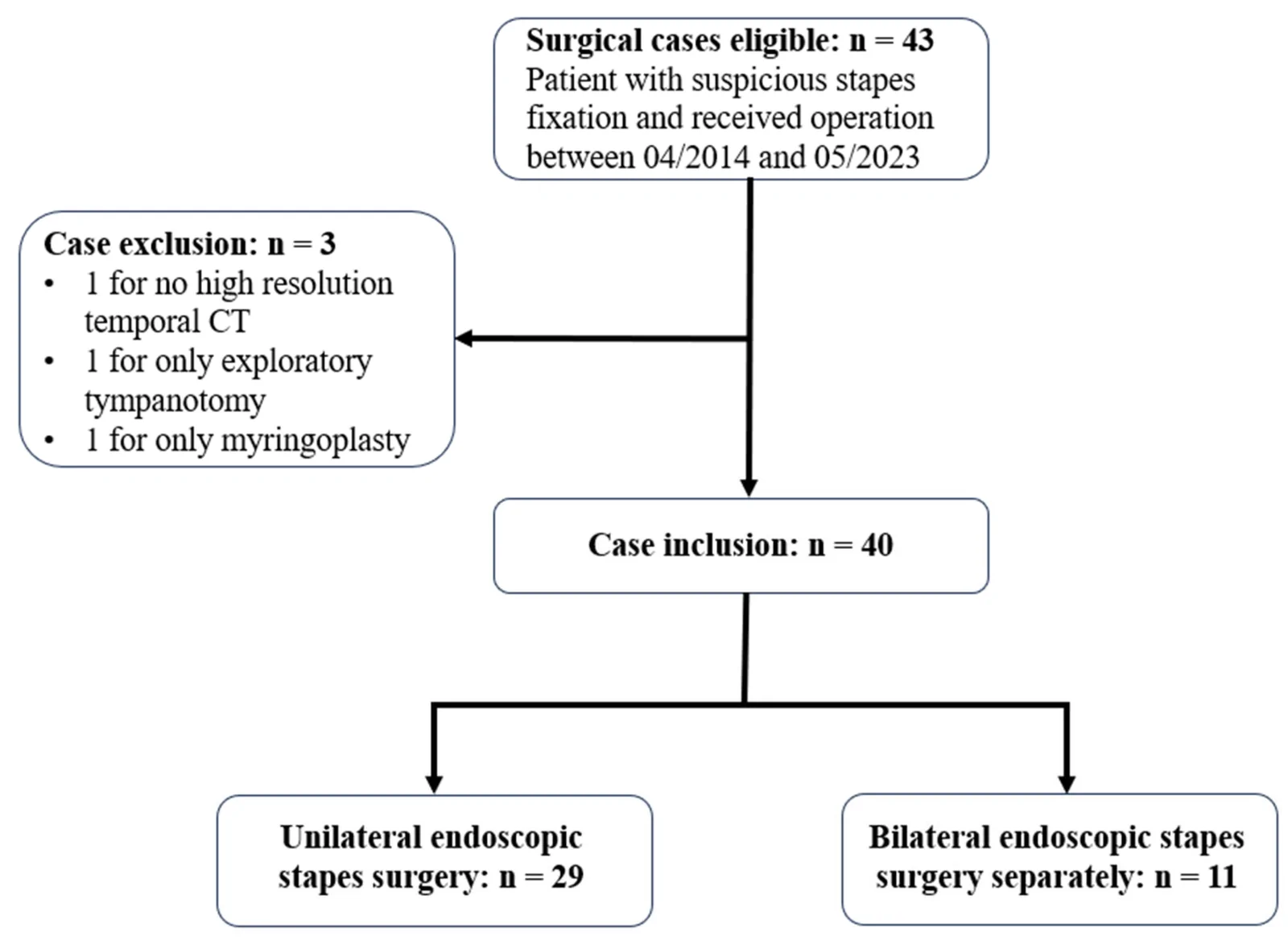
Flow diagram of the inclusion and design process of the study.

A total of 43 patients presenting with suspected stapes disease were enrolled in this study during the specified period. Three cases were excluded: one because of lack of high-resolution temporal bone CT images and the remaining two because the patients only underwent exploratory tympanotomy and myringoplasty (Figure 3).

Among the 40 cases included in the study (9 men [9 ears] and 31 women [42 ears]), the mean age was 46.7 years. In all, 14 patients (35%) underwent surgery on the right ear, 15 (37.5%) on the left ear, and 11 (27.5%) on both ears; a total of 51 ears underwent the procedure. All patients who underwent endoscopic stapes surgery on both ears were women; however, these procedures were performed sequentially rather than simultaneously, with a mean interval of 14.7 months between operations. Otosclerosis was identified in 39 cases (97.5%), and 1 case of chronic otitis media with stapes fixation was observed (Table 1). All patients underwent endoscopic stapes surgery using titanium K-piston prostheses. Most procedures were performed using the stapedotomy technique, whereas a limited number required stapedectomy according to intraoperative findings. Prosthesis diameter was selected intraoperatively according to anatomical requirements.

Postoperative complications were uncommon. One patient experienced transient postoperative dizziness that resolved during follow-up. One patient reported tinnitus at approximately five months after surgery. One patient developed postoperative tympanic membrane perforation. One patient demonstrated postoperative high-frequency air-conduction hearing loss. No profound sensorineural hearing loss, permanent facial nerve paralysis, or other major complications were observed during the follow-up period.

Preoperative PTA revealed a mean bone conduction (BC) threshold of 29.4 dB (standard deviation [SD]: 9.3 dB) and a mean air conduction (AC) threshold of 59.0 dB (SD: 12.5 dB). The mean preoperative ABG was 29.5 dB (SD: 7.9 dB). Postoperatively, the mean BC of PTA was 23.9 dB (SD: 8.6 dB), and the mean AC of PTA was 32.0 dB (SD: 9.6 dB). Furthermore, the mean postoperative ABG was 8.1 dB (SD: 4.6 dB). Significant reductions were observed in BC, AC, and ABG (*p* < 0.0001 at all). The mean ABG closure was 21.4 dB (SD: 7.2 dB), and the postoperative ABG was <20 dB in all cases and <10 dB in 38 ears (74.5%). The median postoperative audiological exam follow-up was 6.8 months. The average operative time was 100 min (SD: 27.6 min; Table 2).

An analysis of the high-resolution temporal bone CT images revealed a mean scutum angle of 70.4° (SD: 7.2°) and a mean EAC cross-sectional area of 0.19 cm^2^ (SD: 0.04 cm^2^).

**Figure 4:**
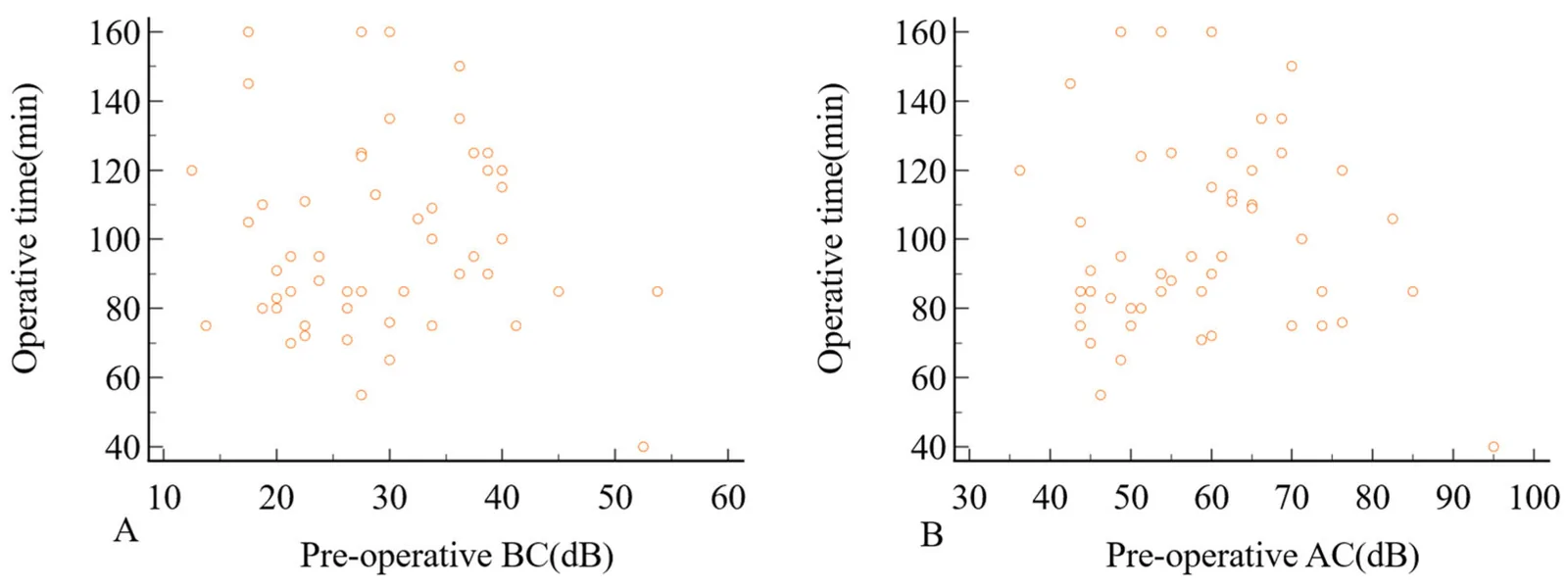

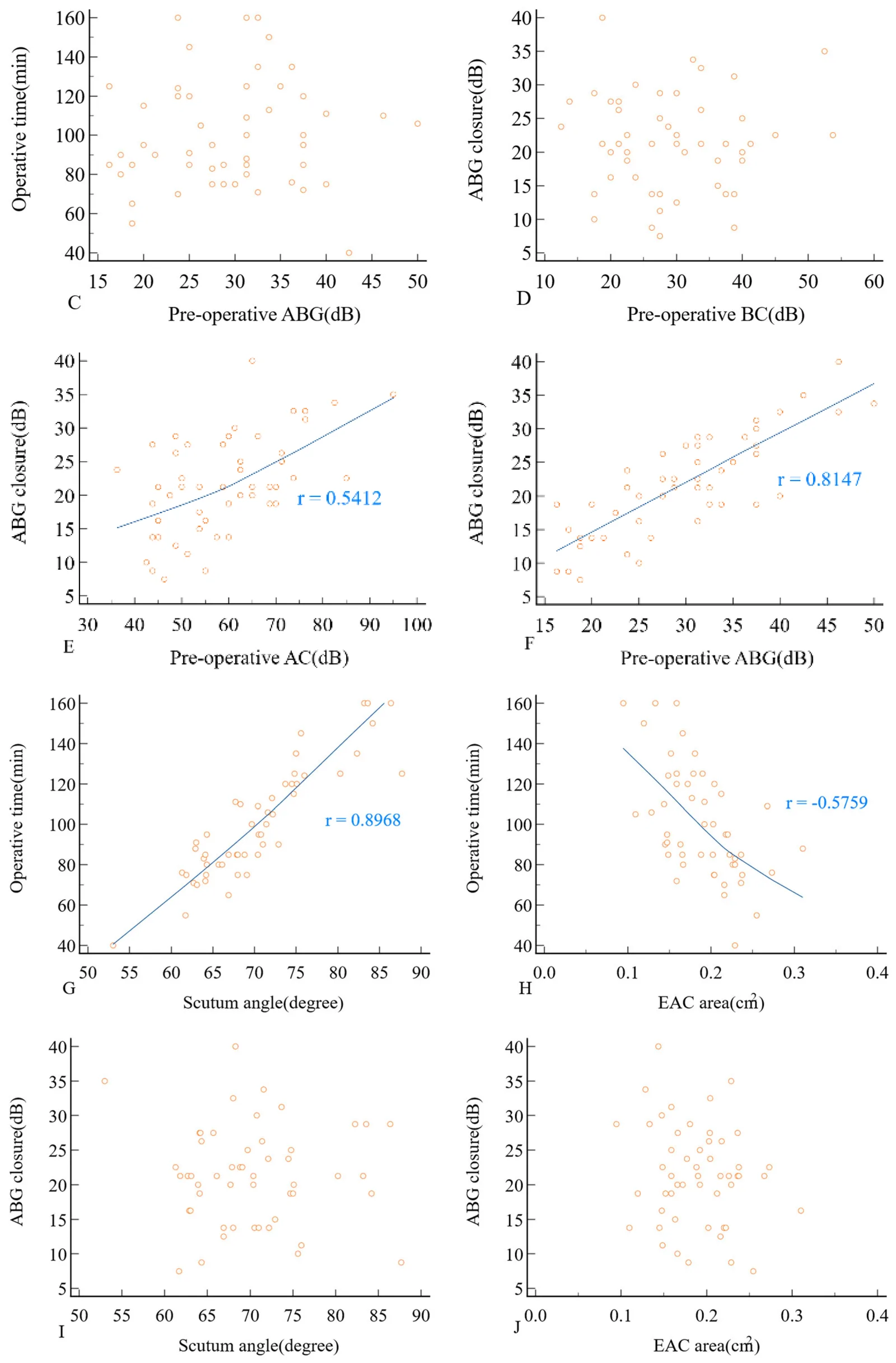
(**A**–**C**) Correlation between operative time and preoperative audiological results. (**D**–**F**) Correlation between air–bone gap (ABG) closure and preoperative audiological results. (**G**) Correlation between operative time and scutum angle. (**H**) Correlation between operative time and external auditory canal (EAC) area. (**I**) Correlation between ABG closure and scutum angle. (**J**) Correlation between ABG closure and EAC area. Note: Correlation coefficients [*r* values] are not presented where no significant correlation was noted.

The results of the correlation analysis revealed very strong positive correlation between operative time and scutum angle (r = 0.8968, *p* < 0.0001). Conversely, moderate negative correlation was observed between operative time and the EAC cross-sectional area (r = −0.5759, *p* < 0.0001). But no correlation was observed between operative time and preoperative BC (r = −0.0613, *p* = 0.6692), AC (r = −0.0235, *p* = 0.8700), or ABG (r = 0.0541, *p* = 0.7063). In addition, ABG closure was positively correlated with preoperative AC (r = 0.5412, *p* = 0.0001) and ABG (r = 0.8147, *p* < 0.0001). However, no correlation was observed between ABG closure and preoperative BC (r = 0.0124, *p* = 0.9314). Moreover, ABG closure exhibited no significant correlation with the EAC cross-sectional area (r = −0.1709, *p* = 0.2306) or scutum angle (r = −0.04765, *p* = 0.7399; Figure 4).

**Figure 5:**
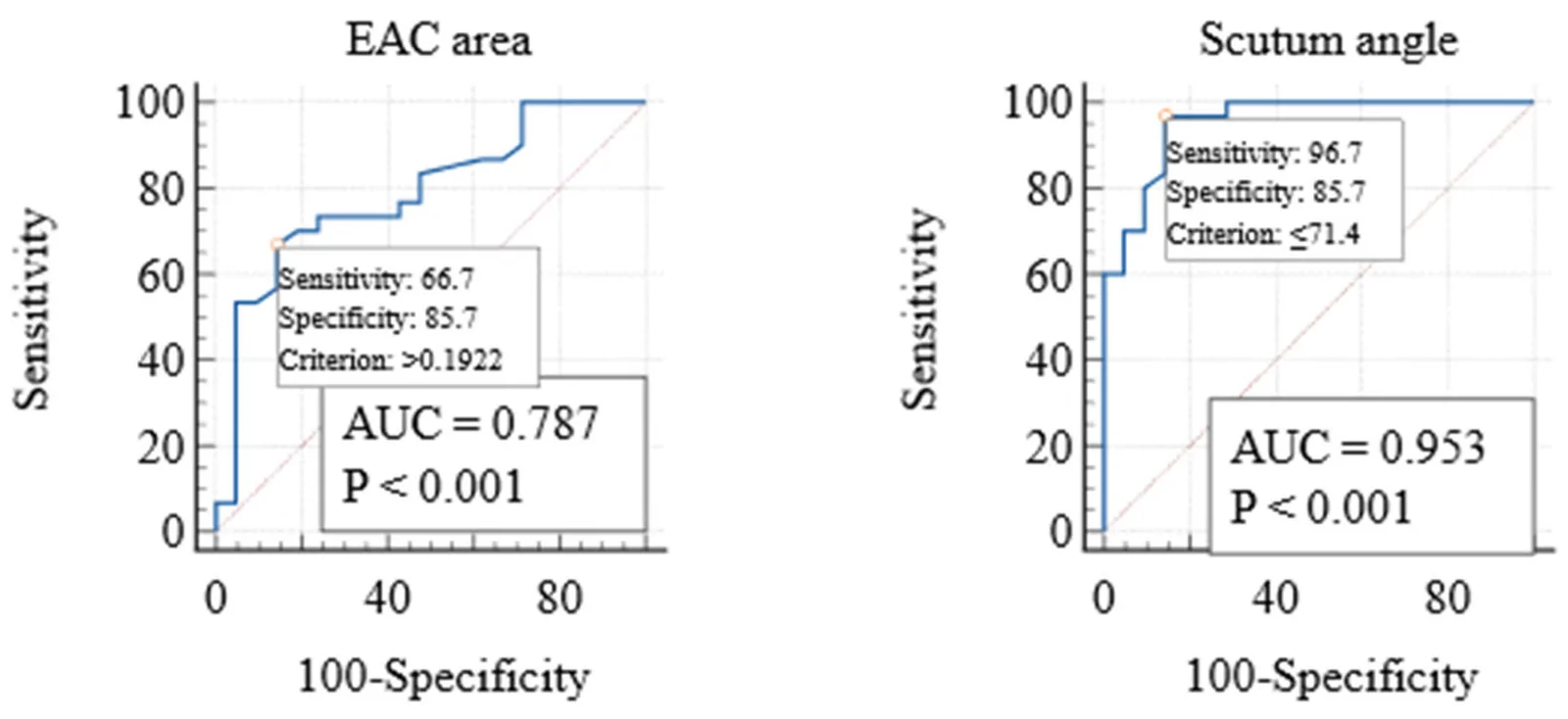
Receiver operating characteristic (ROC) curve analysis of external auditory canal (EAC) area and operative time and of scutum angle and operative time.

Cutoff values were calculated using ROC curve analysis for the cross-sectional area of the EAC and the scutum angle in relation to operative time. For operative time, 0 was used to represent times longer than the average operative time, whereas 1 was used to represent times equal to or less than the average operative time. The ROC curve for the cross-sectional area of the EAC and operative time revealed acceptable discrimination (AUC = 0.787, *p* < 0.0001, sensitivity = 66.67%, specificity = 85.71%), with a cutoff value of 0.1922 cm^2^. Furthermore, the ROC curve for the scutum angle and operative time revealed outstanding discrimination (AUC = 0.953, *p* < 0.0001, sensitivity = 96.67%, specificity = 85.71%) with a cutoff value of 71.4° (Figure 5).

To evaluate whether increasing surgical experience influenced operative time, additional analyses were performed by dividing the study period into three predefined intervals (1 April 2014–31 March 2017, 1 April 2017–31 March 2020, and 1 April 2020–31 March 2023). The mean operative time was 110.0 min (SD: 33.7 min) during the first period (*n* = 15), 94.4 min (SD: 25.5 min) during the second period (*n* = 23), and 98.3 min (SD: 21.5 min) during the third period (*n* = 13). One-way analysis of variance demonstrated no statistically significant difference among the three surgical periods (F = 1.519, *p* = 0.229) (Table 3). Furthermore, linear regression analysis demonstrated no significant temporal trend in operative time (β = −6.16 min per surgical period, 95% confidence interval −16.60 to 4.28; *p* = 0.241).

## 4. Discussion

Advances in endoscopic technology offers high-resolution imaging and superior visualization of structures in the retrotympanum and epitympanum. The rigid endoscope enables comprehensive visualization of target middle ear structures because the angled scope can be rotated, which provides an all-round field of vision [13]. Surmelioglu et al. reported that under endoscopic assistance, the frequency of manipulating and the need for scutum tissue curettage were significantly reduced in stapes surgery. The researchers reported that the incidence of scutum tissue removal was 4.5% in their endoscopic group and 33% in their microscopic group [14]. Kojima et al. demonstrated that endoscopic stapes surgery offers several advantages, including enhanced visualization, improved education for surgical assistants, and lower complication rates (e.g., pain, chorda tympani nerve injury, and ossicular dislocation rates) [12]. Alicandri-Ciufelli et al. presented endoscopic stapes surgery offers several anatomical advantages over the microscopic approach by providing a wide-angle panoramic view of the middle ear and oval window through a transcanal route. These advantages facilitate visualization of critical structures while minimizing the need for canalplasty in appropriately selected patients [11]. Sarkar et al. concluded that endoscopic stapes surgery can be considered an effective alternative to the microscopic technique, particularly in regions with limited resources [15]. However, the endoscopic approach has some disadvantages, including stereoscopic vision absence, working with only one hand, and long learning curve [16].

Despite these disadvantages, endoscopic stapes surgery not only provides excellent visualization and lower complication rates but also yields remarkable audiological outcomes. Daneshi et al. demonstrated that 94.71% of the patients in their endoscopic stapes group achieved postoperative ABG improvement to ≤20 dB and 93.33% of the patients in their microscopic group achieved similar results. There were no significant differences [17]. Furthermore, Migirov et al. reported that at 6 months post-endoscope stapes surgery, the AC and BC thresholds improved from 64 to 29.8 dB and from 30.6 to 25.1 dB, respectively. The average postoperative ABG in that study was <15 dB, with 75% of the patients achieving an ABG of ≤10 dB and 25% achieving an ABG between 10 and 15 dB [18]. Moreover, Sproat et al. revealed that 79% of the patients in their endoscopic and microscopic groups exhibited a postoperative ABG of <10 dB both [19]. Vincent et al. reported that in pediatric and senior patient populations, 93.5% and 94.5% of the patients, respectively, achieved a postoperative ABG of <10 dB [20]. These findings collectively support the safety and efficacy of endoscopic stapes surgery for the surgical management of hearing loss associated with otosclerosis. In the present study, the audiological results revealed significant reductions in BC, AC, and ABG within 2 months following endoscopic stapes operation (*p* < 0.0001 for all). Particularly, the postoperative ABG values for all 51 ears (100%) were below 20 dB, with 38 ears (74.5%) achieving an ABG of <10 dB. These results indicate that surgical intervention led to significant improvement in hearing function. In the analysis of audiological results, ABG closure exhibited a moderate positive correlation with preoperative AC (r = 0.5412) and a very strong positive correlation with preoperative ABG (r = 0.8147). However, no significant correlation was observed between ABG closure and temporal bone CT findings. Koopmann et al. [21] and Sharaf et al. [22] have reported that higher preoperative ABG is associated with greater relative ABG closure. Additionally, Tang reported that reductions in AC were correlated with preoperative AC thresholds [23]. These audiological findings are consistent with the present findings, suggesting that preoperative AC and ABG may serve as predictors of ABG closure following endoscopic stapes surgery.

The operative time for stapes operation has been discussed in several studies, with many suggesting that endoscopic stapes surgery typically results in shorter operative times than does the microscopic approach. This difference is often attributed to the reduced need for removal of bony and soft tissue. Daneshi et al. reported that the operative time was significantly shorter in endoscopic group (31.78 min) than in microscopic group (54.33 min) [17]. Another study conducted in 2017 reported a significantly shorter operative time in endoscopic group than in microscopic group (65.1 min versus 71.2 min) [14]. However, one study has indicated no significant difference in operative time between the two techniques, with reporting average operative time of 45.1 ± 8.4 min for endoscopic group and one of 48.7 ± 5.6 min for microscopic group [24]. Additionally, some studies have reported the mean operative time to be significantly longer in endoscopic group [25,26]. This discrepancy in findings can be attributed to two main factors: the additional time required for frequent wiping of blood-stained endoscope and the need to achieve hemostasis with only one hand. Another contributing factor is the long learning curve associated with mastering endoscopic surgery.

In Kuo et al., the EAC width on temporal bone CT scans had only a weak correlation with operative time (r = 0.07) [25]. Although anatomical constraints of the external auditory canal and scutum morphology have been discussed in both microscopic and endoscopic stapes literature, quantitative CT-based analysis of these parameters specifically in relation to operative time under an exclusively endoscopic approach remains limited. The present study should therefore be viewed as a refinement and quantitative expansion of existing anatomical observations rather than the introduction of a novel surgical concept. The present study examined temporal bone CT findings to assess their potential impact on both audiological outcomes and operative time. The EAC cross-sectional area and scutum angle were analyzed, and the results revealed that neither the EAC area nor the scutum angle was significantly correlated with audiological outcomes. However, operative time had a moderate negative correlation with the EAC area (r = −0.5759) and a very strong positive correlation with the scutum angle (r = 0.8968). A larger EAC area may provide more surgical space, which can reduce the need to sacrifice the scutum or other structures to obtain clear surgical field. Additionally, a lower scutum angle enables the endoscope to approach the tympanic sinus more easily, thereby reducing the need to remove neighboring structures. The current findings have several potential clinical implications. Patients with a relatively smaller EAC cross-sectional area and larger scutum angle are more likely to require additional scutum curettage to achieve adequate visualization of the oval window during endoscopic surgery. Consequently, these anatomical characteristics may increase operative complexity and prolong operative time. Preoperative identification of these anatomical features may therefore facilitate surgical planning by allowing surgeons to anticipate technical difficulty, prepare appropriate surgical instruments, counsel patients regarding operative complexity, and determine whether an exclusively endoscopic approach is appropriate in selected cases. Such preoperative assessment may also reduce the likelihood of unexpected intraoperative conversion to microscopic surgery.

ROC curve analysis of the EAC area and operative time as well as of the scutum angle and operative time revealed cutoff values of 0.1922 cm^2^ (AUC = 0.787, *p* < 0.0001) and 71.4° (AUC = 0.953, *p* < 0.0001), respectively. Although ROC curve analysis identified cutoff values for the EAC cross-sectional area and scutum angle, these thresholds should be interpreted with caution. They were derived from a relatively small retrospective cohort at a single institution and have not undergone external validation. Therefore, these cutoff values should be regarded as exploratory findings that generate hypotheses for future investigation rather than definitive criteria for clinical decision-making.

Because this study spanned approximately nine years, the potential influence of increasing surgical experience was considered. Additional analyses were therefore performed by dividing the study period into three predefined intervals. Neither one-way analysis of variance nor linear regression analysis demonstrated a significant association between surgical period and operative time. These findings suggest that the observed relationships between CT-derived anatomical parameters and operative time were unlikely to be primarily attributable to progressive surgical experience during the study period.

This study has several limitations should be acknowledged. First, the study did not include a control group, and therefore, no precise comparison between endoscopic and microscopic approaches could be conducted. Second, because this was a retrospective study with a small sample size, we were unable to gather complete data on patient characteristics that may influence operative time. The small sample size also limited the precision of our estimates. Third, although bilateral procedures were included, the operations were performed sequentially with independent preoperative assessment and postoperative follow-up for each ear. Nevertheless, residual within-patient correlation cannot be completely excluded. Fourth, all CT measurements were performed by a single observer using a standardized measurement protocol. Although this approach minimized interobserver variability, formal assessment of intraobserver reproducibility was not performed. Finally, the proposed ROC-derived anatomical thresholds have not been externally validated and should therefore be interpreted as exploratory. Despite these limitations, this study provides valuable insights into the suitability and possible predictors of endoscopic stapes surgery.

Future prospective multicenter studies with larger patient populations are warranted to validate the proposed anatomical thresholds and determine whether preoperative CT-based assessment can be incorporated into routine surgical planning for endoscopic stapes surgery. In addition, future investigations should evaluate intraobserver and interobserver reproducibility of CT measurements and explore whether automated image analysis techniques may further improve measurement reliability.

## 5. Conclusions

This study demonstrates that endoscopic stapes surgery achieves significant audiological improvement in patients with otosclerosis. Preoperative ABG and AC levels were associated with magnitude of postoperative ABG closure. A smaller EAC cross-sectional area and a larger scutum angle on preoperative temporal bone CT were associated with prolonged operative time in exclusive endoscopic stapes surgery, whereas these anatomical parameters were not associated with postoperative hearing outcomes. Preoperative CT evaluation may assist surgeons in anticipating operative complexity and planning the most appropriate surgical approach. However, the proposed anatomical thresholds require prospective external validation before routine clinical application.

## Figures and Tables

**Table 1:** Basic characteristics of patients who underwent endoscopic stapes surgery.

Characteristic		Cases (*n* = 40)
Sex	Male	9 (22.5%)
	Female	31 (77.5%)
Age (years)	Mean	46.7
	Range	13–67
Side of ear that underwent operation	Right	14 (35%)
	Left	15 (37.5%)
	Bilateral	11 (27.5%)
Reason for operation	Otosclerosis	39 (97.5%)
	Chronic otitis media with stapes fixation	1 (2.5%)
Interval between bilateral ear operation (months)	Mean	14.7
	Range	4.9–63.7

**Table 2:** Preoperative and postoperative audiological results, temporal bone CT findings, and operative time.

	All Ears (*n* = 51)		
Bone conduction ± SD (dB)	Preoperative	29.4 ± 9.3	
	Postoperative	23.9 ± 8.6	*p* < 0.0001
Air conduction ± SD (dB)	Preoperative	59.0 ± 12.5	
	Postoperative	32.0 ± 9.6	*p* < 0.0001
ABG ± SD (dB)	Preoperative	29.5 ± 7.9	
	Postoperative	8.1 ± 4.6	*p* < 0.0001
Postoperative ABG (%)	≤10 dB	74.5	
	≤20 dB	100	
ABG closure ± SD (dB)		21.4 ± 7.2	
Scutum angle ± SD (degree)		70.4 ± 7.2	
EAC cross-sectional area ± SD (cm^2^)		0.19 ± 0.04	
Operative time ± SD (minute)		100 ± 27.6	

SD, standard deviation; ABG, air-bone gap; EAC, external auditory canal.

**Table 3:** Operative time according to surgical period.

Surgical Period	*n*	Operative Time (min)	*p* Value
1 April 2014–31 March 2017	15	110.0 ± 33.7	
1 April 2017–31 March 2020	23	94.4 ± 25.5	
1 April 2020–31 March 2023	13	98.3 ± 21.5	
One-way ANOVA			*p* = 0.229

## Data Availability

The data that support the findings of this study are available from the corresponding author upon reasonable request.

## Abbreviations

The following abbreviations are used in this manuscript:

EAC	External auditory canal
ABG	Air–bone gap
ROC	Receiver operating characteristic
PTA	Pure tone audiometry
CT	Computed tomography
BC	Bone conduction
AC	Air conduction
SD	Standard deviation
